# Similarities in Perceived Marital Responsiveness Among Arab Couples After Retirement

**DOI:** 10.1093/geroni/igaf122.133

**Published:** 2025-12-31

**Authors:** Reem Nashef-Hamuda, Dikla Segel-Karpas, Roi Estlein, Yuval Palgi

**Affiliations:** University of Haifa, Haifa, HaZafon, Israel; University of Haifa, Haifa, HaZafon, Israel; University of Haifa, Haifa, HaZafon, Israel; University of Haifa, Haifa, HaZafon, Israel

## Abstract

This study explores the intricate relationship between perceived marital responsiveness, life satisfaction, and marital quality among retired Arab couples—a context largely understudied in non-Western collectivist societies. A total of 108 retired heterosexual couples (216 participants; husbands’ mean age = 70.31 years, wives’ mean age = 65.07 years, mean marital duration = 43.58 years) completed surveys assessing perceived marital responsiveness, life satisfaction, and marital quality. Using Dyadic Response Surface Analysis (DRSA), this study examined both individual and dyadic effects of perceived responsiveness on partners’ life satisfaction and marital quality. Contrary to prior research, dyadic congruence in perceived responsiveness—the degree to which partners perceive responsiveness similarly—did not significantly predict life satisfaction or marital quality. Instead, individual perceptions of responsiveness emerged as a stronger predictor of both outcomes, highlighting the centrality of personal emotional experiences over dyadic similarity in shaping well-being in later life. Drawing upon an integrative Family Systems and Socioemotional Selectivity framework, these findings challenge Western-centric assumptions about marital dynamics in aging couples and underscore the cultural nuances of emotional support in Arab society. The study contributes to a more nuanced understanding of marital resilience after retirement, with implications for relationship interventions, aging policy, and cross-cultural gerontological research.

